# On Missingness Features in Machine Learning Models for Critical Care: Observational Study

**DOI:** 10.2196/25022

**Published:** 2021-12-08

**Authors:** Janmajay Singh, Masahiro Sato, Tomoko Ohkuma

**Affiliations:** 1 Fuji Xerox Co, Ltd Yokohama Japan

**Keywords:** electronic health records, informative missingness, machine learning, missing data, hospital mortality, sepsis

## Abstract

**Background:**

Missing data in electronic health records is inevitable and considered to be nonrandom. Several studies have found that features indicating missing patterns (missingness) encode useful information about a patient’s health and advocate for their inclusion in clinical prediction models. But their effectiveness has not been comprehensively evaluated.

**Objective:**

The goal of the research is to study the effect of including informative missingness features in machine learning models for various clinically relevant outcomes and explore robustness of these features across patient subgroups and task settings.

**Methods:**

A total of 48,336 electronic health records from the 2012 and 2019 PhysioNet Challenges were used, and mortality, length of stay, and sepsis outcomes were chosen. The latter dataset was multicenter, allowing external validation. Gated recurrent units were used to learn sequential patterns in the data and classify or predict labels of interest. Models were evaluated on various criteria and across population subgroups evaluating discriminative ability and calibration.

**Results:**

Generally improved model performance in retrospective tasks was observed on including missingness features. Extent of improvement depended on the outcome of interest (area under the curve of the receiver operating characteristic [AUROC] improved from 1.2% to 7.7%) and even patient subgroup. However, missingness features did not display utility in a simulated prospective setting, being outperformed (0.9% difference in AUROC) by the model relying only on pathological features. This was despite leading to earlier detection of disease (true positives), since including these features led to a concomitant rise in false positive detections.

**Conclusions:**

This study comprehensively evaluated effectiveness of missingness features on machine learning models. A detailed understanding of how these features affect model performance may lead to their informed use in clinical settings especially for administrative tasks like length of stay prediction where they present the greatest benefit. While missingness features, representative of health care processes, vary greatly due to intra- and interhospital factors, they may still be used in prediction models for clinically relevant outcomes. However, their use in prospective models producing frequent predictions needs to be explored further.

## Introduction

### Background

The increasing availability of electronic health record (EHR) data collected from hospitals, especially from their intensive care units (ICU), has encouraged the development of various models for disease diagnosis [1-4]. Machine learning and specifically deep learning models, given their ability to adequately learn nonlinear representations and temporal patterns from large amounts of data, have been widely applied to capture complex physiological processes, and several works have demonstrated their usefulness [5]. Most works use retrospective observational data to train supervised models for a variety of clinically important outcomes like mortality or sepsis. Some more recent works have also developed models more suited to actual clinical needs by evaluating models prospectively and using early warning scores as baselines [6]. Models used to learn human physiological processes from EHRs tackle intrinsic problems in health care data, particularly that of irregular sampling and large amount of missing information [7].

Several methods have been developed to handle the inevitably large amount of missing data in EHRs. Simpler methods like incomplete record deletion (also called complete case analysis) propose to simply delete those records where any value is missing. Various imputation techniques ranging from simple mean imputation to sophisticated methods like multiple imputation with chained equations are also commonly used [8]. More recently, deep learning models have been proposed to learn the underlying process generating the data as a method for better inferring missing values [9]. A consensus regarding a best universal model to handle missing data does not exist in literature, and it is generally understood to depend heavily on the task and the nature of the data itself. However, a returning consideration in all studies on missing data is the nature of missingness. In Rubin [10], missing data were classified into 3 categories: missing completely at random, missing at random, and missing not at random. The nature of missingness in EHRs has been generally understood to belong to the last category, missing not at random [11]. This means that missing values cannot be inferred using observed values, subjecting all methods to problems of bias.

Considering the inevitability of bias, methods seek to minimize it by considering imputed value uncertainty or developing more sophisticated processes to learn underlying distributions [8,12]. A returning simple yet effective motif in deep learning models for EHRs is to use informative missingness (IM) features. First introduced in Lin and Haug [11], the method has repeatedly been shown to improve performance of health care models for a variety of outcomes [13-16]. A particularly efficient use was demonstrated in Lipton et al [13], where simply augmenting zero-imputed data with corresponding binary missingness indicators greatly improved over the baseline model. The basic assumption underlying the use of IM features is that the inclusion of health care process variables like laboratory tests conducted or drugs prescribed provides important information about the state and evolution of a patient’s health. These variables are usually inputted to the model as binary indicators of observation/missingness, but some studies have also propounded modifying or augmenting this representation to include additional information such as time since last observation [17,18]. We use the term health care process variables interchangeably with IM features.

This use of health care process variables as feasible features to model patient health is supported by studies spanning several decades and countries, indicating that test ordering behavior and drug prescriptions are associated with the underlying pathology. For example, Kristiansen et al [19] established that the medical condition at hand was the strongest determinant of test ordering behavior, and Weiskopf et al [20] and Rusanov et al [21] found a statistically significant relationship between data completeness and patient health status, finding that those susceptible to adverse outcomes have more information collected. A recent study also highlighted that EHR data are observational and display a patient’s interactions with the health care system and thus any information from there can only serve as a proxy measure of the patient’s true state [22]. They further found that the presence of laboratory test orders, regardless of other information like numerical test values, had a significant association with odds of 3-year survival. This suggests that laboratory test orders encode information separately from laboratory test results, as corroborated by Pivovarov et al [23].

Despite improvements in model performance on including IM features, their use is considered to have limited applicability. Missing information may occur due to several factors, not all which pertain to patient pathology or a physician’s mental model of the diagnosis process. Within a hospital, some tests may be conducted following general guidelines or as standard practice for all patients regardless of underlying condition [23]. Physicians also vary by years of experience and attitudes in coping with uncertainty, which has been shown to affect test ordering behavior [24]. In addition, variations between hospitals as test ordering may depend on resource constraints and variations due to geographic separation as ICU case-mix changes are further exacerbated when making international comparisons [25,26]. And while machine learning models rely on improved performance on chosen metrics as a justification for continued use of IM features, evaluation has mostly been on single-center data under retrospective task settings. Even where multicenter data are used, hospitals are often not geographically distinct, preventing the assessment of model generalization to different demographic mixes and practices. Also, only recently have some works evaluated their models prospectively, better reflecting real-world clinical utility, but evaluation metrics differ across studies, some choosing to use the concordance index (also called the area under receiver operating curve [AUROC]) while others prefer the area under precision recall curve [27,28].

The ways in which use of IM features is supported and challenged creates an apparent disjunction and casts doubts on their true usefulness. This was perhaps exemplified in the PhysioNet 2019 Challenge [29] for early prediction of sepsis, which saw many submissions using some modification of IM features [16-18,30,31]. The challenge was designed to evaluate models on prospective prediction performance and used datasets from 3 geographically distinct hospital systems, one of which was never provided to the participants. While several models had reasonable performance on hospitals they had at least partial access to, scores dropped substantially on the third, unseen hospital. Models using more sophisticated modifications of IM features saw a larger drop than those using simple binary variables or no representation of health care processes.

### Objectives

In this study we seek to empirically verify and understand the effect that including IM features has on health care machine learning models. We selected 3 common outcomes of interest, mortality, length-of-stay, and sepsis, and trained models for 2 task settings. The first, shared by all outcomes, is entire record classification where the model provides a prediction at the end of a patient’s ICU stay. The second is hourly prediction of label, and only the sepsis label is used for this task.

We verify the effect of IM feature inclusion on performance, generalizability, and clinical utility of models in 3 steps. First, to get a comprehensive understanding of model performance, binary classification models for each of the outcomes were trained and evaluated using multiple metrics. Since class imbalance varies between outcomes, we could also evaluate model robustness. Second, for the sepsis outcome, since data from 2 distinct hospital systems were available, we could evaluate model generalizability and test whether that is affected by IM features. Third, again for the sepsis outcome, since labels for every hour of patient data were available, we trained a model for temporal prediction of sepsis. We evaluated this model on the hidden hospital system’s data in a simulated prospective manner, in the process understanding how the models would behave in an actual clinical setting and what differences in performance can be expected by including IM features.

Finally, we hypothesized that health care processes vary across patient demographics and ICU types, which may result in varying missingness rates and patterns across subgroups. Previous works have shown how laboratory variation (and thus test ordering behavior) may vary based on these criteria; this was also seen in our data analysis [32,33]. Thus, we were motivated to see model performances for different subgroups, as well as to study the different extent to which IM features improve model performance within a subgroup. Based on our data analysis, age and ICU type subgroups were chosen. Since testing was also done on the hidden hospital, we could see how generalization on subgroups is affected by including IM. We could also verify whether models can use IM features to capture the relationship between test ordering and patient pathophysiology despite intra- and interhospital variations.

## Methods

In this section we describe the datasets used for this study and the preprocessing pipeline. We also describe how outcomes of interest were defined. This is followed by an overview of the task settings and experiments with model implementation details.

### Datasets

Data from the PhysioNet 2012 and 2019 Challenges were used for this study. From the PhysioNet 2012 [34] dataset (P12), we used patient records from training set A and open test set B, each consisting of data from 4000 patients collected from 4 types of ICUs. Several patient outcomes are provided of which we selected in-hospital death (mortality) and length of stay (number of days between patient’s admission to the ICU and end of hospitalization, LOS). We binarized the LOS outcome setting as 3 days as a heuristic decision threshold, similar to previous studies [14]. The data consist of static patient descriptors as well as temporal variables representing patient vitals (low missingness) and values from laboratory tests conducted (high missingness). Imbalance ratios of mortality and LOS were different, at 13.9% and 6.5%, respectively, for set A and 14.2% and 7.0%, respectively, for set B. Since P12 was extracted from the MIMIC II (Multiparameter Intelligent Monitoring in Intensive Care) Clinical Database [35], the data were from one hospital system only.

The PhysioNet 2019 [29] dataset (P19) comprised patient records from 3 geographically distinct US hospital systems. A total of 40,336 patient records, 20,336 from hospital A (set A) and 20,000 from hospital B (set B), from 2 ICU types were used. Data from hospital C were not available for download. Since the challenge was aimed at model development for early prediction of sepsis, a corresponding binary label is provided for every hour of the patient’s record. Labeling was done in accordance with the Third International Consensus Definitions for Sepsis and Septic Shock (Sepsis-3) criteria [36]. It is important to note that to facilitate training models for early prediction, patients who eventually developed sepsis were labeled as such starting 6 hours before a confirmed diagnosis. More details about the definition of the sepsis label may be found in Multimedia Appendix 1. Available variables in the dataset are similar to that in P12, describing static as well as temporal patient features with varying missingness. The cohort from hospital A consisted of 8.8% of patients who developed sepsis while it was 5.7% for hospital B. Due to the cohort selection procedure followed by Reyna et al [29], few patients have sepsis from the start of ICU admission. Only 2.2% of hourly records for hospital A and 1.4% for hospital B are labeled as corresponding to sepsis. For analysis of the extent of missingness in the various datasets, please see Figures S1-S5 in Multimedia Appendix 2.

### Preprocessing

Data preprocessing was done using a similar pipeline as described in multiple previous studies [13,14,37]. Data from P12 were resampled on an hourly basis, while P19 data were already resampled. While resampling, some patient records were found to have static descriptors only and others had missing outcome labels in both sets of P12. These were removed, leaving 3997 patient records in set A and 3993 in set B. Invasive and noninvasive measurements of the same variable present in P12 were averaged to form aggregate measurements. In P19, end tidal carbon dioxide was a variable observed in only hospital B, so it was removed from consideration. Static patient features describing age, gender, or ICU type identifiers were not used as inputs. This left us with 33 features in P12 and 34 in P19, which were used for model training. To deal with missing data, zero imputation was performed in both datasets, since Lipton et al [13] showed that this simple strategy proved quite effective when used to train deep learning models.

For model training and evaluation, training and testing sets were identified. Set A from both datasets was used for training while set B was shown to the model only for final evaluation. It is worth noting again that set B in P19 belonged to a distinct hospital system. Data were standardized before inputting to the model. Mean and variance from training data were used to standardize corresponding test data.

Finally, we describe the derivation of features to represent missingness. We selected the simplest representation using binary indicator variables, with a 1 used to denote variable observation and a 0 otherwise. Every feature described earlier had a corresponding missingness indicator that was appended to the feature vector as in Lipton et al [13]. This resulted in 66 features for P12 and 68 for P19.

### Modeling Methodology

Since patient pathophysiology evolves nonlinearly over time, sequential models like recurrent neural networks (RNN) are considered suitable and have often been used in previous works [38]. We used a gated RNN variant, specifically a gated recurrent unit (GRU) to model long EHR sequences [39]. A multilayer perceptron followed by a sigmoid layer were used after the GRU to output binary label probabilities.

The model was implemented in Pytorch [40] and trained using minibatch gradient descent to minimize binary cross entropy loss with Adam [41] as the optimizer. Models trained with IM augmented features are denoted by masking while those trained with patient physiological features only are denoted by no masking.

We performed 5-fold stratified cross validation for hyperparameter tuning and to prevent model overfitting. To tune hyperparameters, we performed an iterative ranging investigation to determine a suitable grid followed by a grid search [42]. Maximum averaged AUROC and utility score across all folds were chosen as the criteria for hyperparameter set selection for the retrospective and simulated prospective tasks, respectively [29]. No attempt was made to tune model architecture as our focus was not to propose a new model but to evaluate IM feature effectiveness.

### Task Settings

We analyzed the effectiveness of including IM features by defining 2 tasks, (1) retrospective classification where we verify IM usefulness on model performance, calibration, and generalizability and (2) simulated prospective classification to study IM effect on model prediction trends in a temporal manner.

#### Retrospective Classification

In this setting, the model is trained to predict the appropriate label at the end of a patient’s hospital stay. For this purpose, mortality and LOS labels were used directly from the outcomes provided in P12. For P19, a sepsis-overall label was derived from the hourly labels provided. If a patient developed sepsis at any time, their entire record was marked as positive for sepsis. The task for all 3 labels was binary classification after using the entire patient record as input. We studied the effect of IM in 2 steps, overall classification and subgroup analysis:

- To verify changes in performance on IM inclusion, the models were evaluated on all of the testing data for all datasets and labels. Multiple evaluation metrics were used to understand how IM features change performance and calibration while data from a distinct hospital were used to evaluate changes in model generalizability.
- To study extent of improvement on different patient subgroups, models were trained on all of the training data (representative of a general ICU population) and evaluated on identified subgroups made from the test set. Both datasets provided 3 general patient descriptors: age, gender, and ICU type. Visual comparison of variable observation differences between these strata was performed. Gender showed no substantial difference in variable observation. Different ICU types displayed clear differences as did age after binning into suitable intervals (Figures S6-S11 in Multimedia Appendix 2). These strata were chosen for subgroup analysis.

#### Simulated Prospective Classification

Only P19 was used for this task since P12 did not have hourly labels. The model was trained to predict patient probability of sepsis at every hour using the shifted labels provided in the dataset. At time *t*, information from the beginning of the patient record to *t* was used to make a prediction. This ensured prospective usefulness of the model. Since the model was trained on labels shifted by 6 hours (for septic patients), we expected the model to learn early signs of sepsis onset. The sepsis-overall label described earlier was used for cross-validation and hyperparameter tuning.

### Performance Evaluation

Model discriminative ability was judged by the concordance index or AUROC. Since this is known to be an over optimistic measure for imbalanced datasets [43], we also use the precision-recall curve and average precision to evaluate predictive value [44,45]. Finally, 2 measures were used to assess model calibration: reliability plots and Brier score. The former was useful to visualize calibration changes against different levels of model uncertainty. The latter was used to quantify an averaged deviation from true probabilities and as a convenient summary of uncertainty, resolution, and reliability [46]. We also visualized the number of samples in each bin of the reliability plots by varying marker area proportional to the squared root of the bin size scaled by a constant factor. Finally, AUROC and Brier score were reported with 95% confidence intervals computed with 10,000 bootstrap replications to obtain a good estimation of model performance up to the second significant digit [47].

## Results

### Retrospective Classification

#### Overall Classification

**Figure 1:**
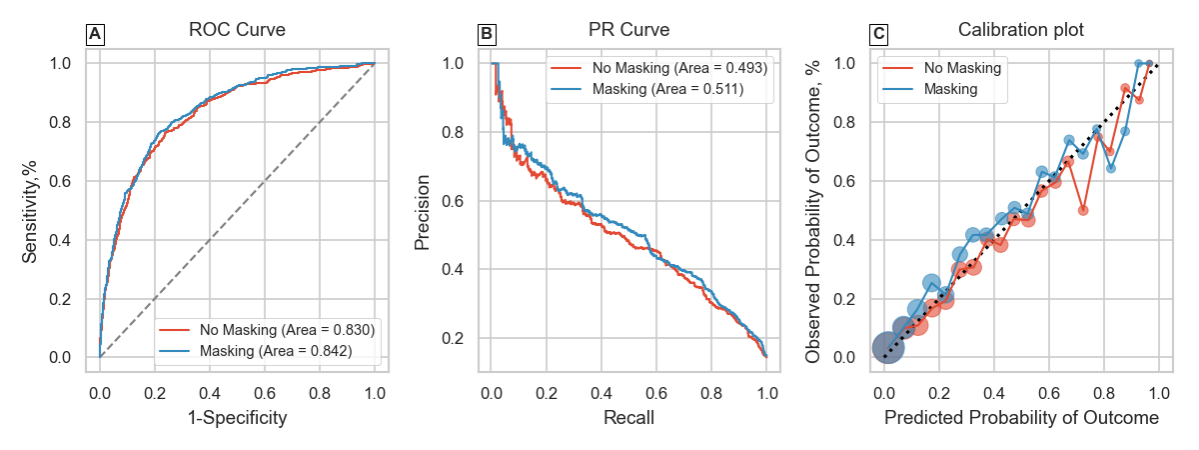
Receiver operating characteristic (ROC) curve, precision-recall (PR) curve, and calibration plot for the PhysioNet 2012 Challenge mortality classification task.

**Figure 2:**
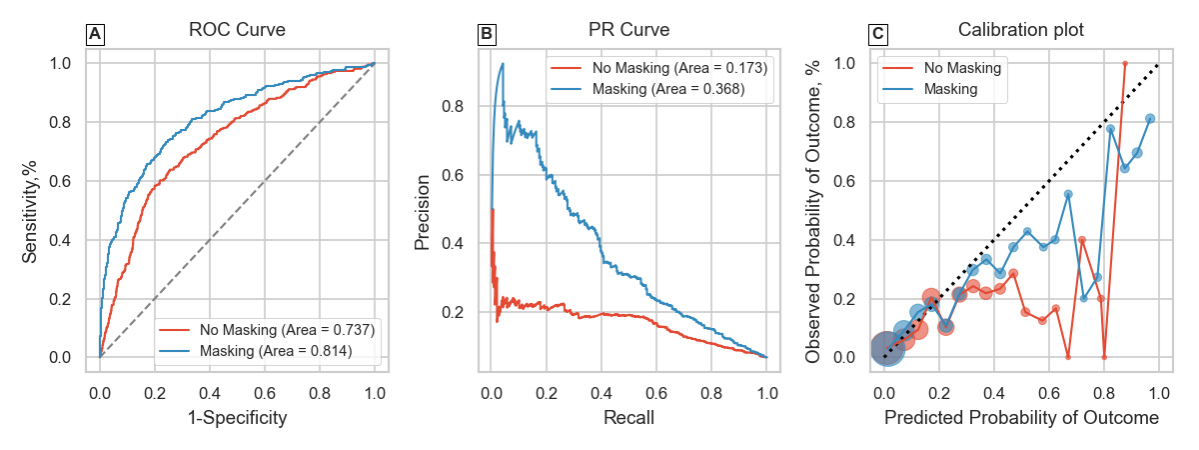
Receiver operating characteristic (ROC) curve, precision-recall (PR) curve, and calibration plot for the PhysioNet 2012 Challenge length of stay classification task.

**Figure 3:**
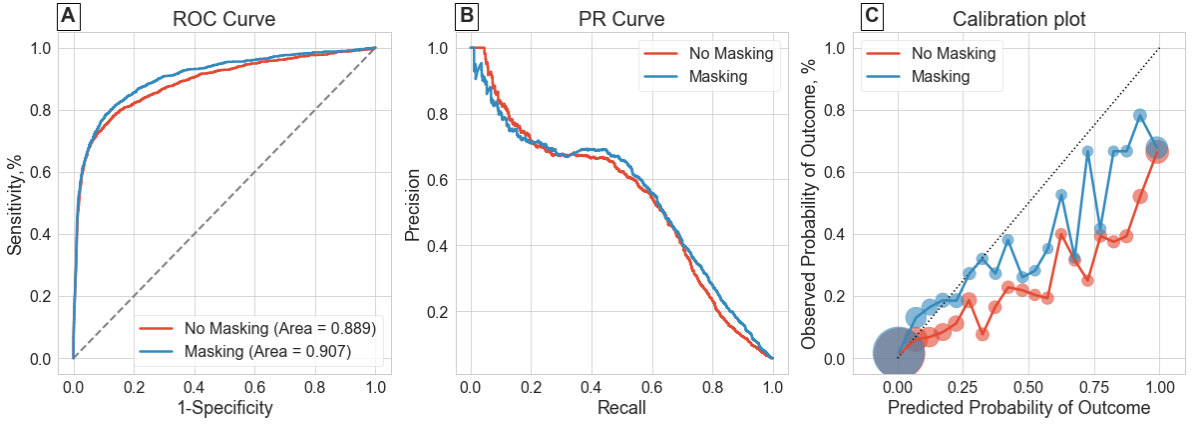
Receiver operating characteristic (ROC) curve, precision-recall (PR) curve, and calibration plot for the PhysioNet 2019 Challenge sepsis-overall classification task.

The first 3 rows of Table 1 summarize results for the overall classification tasks. Including IM resulted in considerable improvements over using patient physiological features only for both tasks on P12 and the sepsis-overall task on P19. The extent of improvement in average precision mimicked trends of improvements in AUROC. The no masking model had an average precision of 0.493 on the P12 mortality task, and including IM features improved this to 0.511. The performance gain was more marked for the P12 LOS task, as average precision was 0.173 without and 0.368 with masking. It is worth noting that the derived LOS label in P12 had higher class imbalance than the mortality label for the same dataset. The P19 sepsis-overall task also saw an improvement in average precision where the no masking model achieved 0.537 and this was 0.547 for the masking model. Panels A and B of Figures 1-3 graphically show the receiver operating characteristic and PR curves for these tasks.

Including IM features also improved model calibration scores in all 3 cases, as seen by the Brier score (lower is better). The improved Brier scores (0.039 with IM features vs 0.045 without) for the P19 sepsis-overall task where evaluation was on a distinct hospital suggests that the model does not overfit to hospital-specific health care process variables. Examining panel C of Figures 1-3 shows the calibration plots for each task setting. The 2 models had very similar plots for the P12 mortality task. The difference was again most pronounced for the P12 LOS task, where the masking model had better calibration at higher model certainties (predicted probabilities). The masking model also showed improved calibration for the P19 sepsis-overall task seen in Figure 3C.

**Table 1:** Results of model discrimination and calibration for all task settings on the test data. These correspond to internal validation for PhysioNet 2012 Challenge and external for PhysioNet 2019 Challenge.

	Masking (AUROC^a^), mean (SD)	Masking (Brier), mean (SD)	No masking (AUROC), mean (SD)	No masking (Brier), mean (SD)
P12^b^ mortality	0.842 (0.82-0.86)	0.093 (0.087-0.100)	0.830 (0.81-0.85)	0.095 (0.088-0.101)
P12 LOS^c^	0.814 (0.79-0.84)	0.054 (0.049-0.060)	0.737 (0.71-0.77)	0.064 (0.058-0.070)
P19^d^ sepsis-overall	0.907 (0.90-0.92)	0.039 (0.036-0.041)	0.889 (0.88-0.90)	0.045 (0.043-0.048)
P19 sepsis-frequent	0.757 (0.74-0.77)	0.014 (0.013-0.014)	0.766 (0.75-0.78)	0.014 (0.013-0.015)

^a^AUROC: area under the curve of the receiver operating characteristic.

^b^P12: PhysioNet 2012 Challenge.

^c^LOS: length of stay.

^d^P19: PhysioNet 2019 Challenge.

#### Subgroup Analysis

Tables 2-4 summarize model performances on the identified subgroups for the 3 overall classification task settings. For variance estimation in results, the subgroup data were bootstrapped keeping the sample size equal to subgroup size. These results have also been visualized as bar plots in Figures S12-S14 in Multimedia Appendix 2.

For the P12 mortality task in Table 2, the no masking model outperformed the masking model for the age bins 35 years and younger and 45 to 55 years, while the masking model had better performance for all other age groups. The best AUROC over all ages was achieved by the masking model on the 35- to 45-year group, which also saw the largest improvement on including IM features (2.6%). While younger and middle-aged groups saw inconsistent performance changes on IM inclusion, older patients (older than 55 years) showed consistent improvements from 0.8% to 1.5% in all-cause mortality classification. When considering performances in different ICUs, the masking model generally had better performance except for the coronary care unit (CCU), but the difference was not substantial. The cardiac surgery recovery unit saw the highest AUROC and also the greatest improvement of 1.7% on IM inclusion.

Similar to the prominent improvements in the P12 LOS-overall classification task, the masking model considerably outperformed the no masking model for all age and ICU type subgroups. The youngest age group, 35 years and younger, saw an improvement of 15.5% in AUROC, becoming the subgroup with the best performance out of all age groups. Comparatively, the 55- to 65-year subgroup, which had the best model performance without IM, saw an improvement only of 0.7%. The cardiac surgery recovery unit again saw the largest performance gain on IM inclusion, of 13.1%, followed by the surgical ICU with 10.2% and the CCU, with a relatively small gain of 2.8%.

Finally for the P19 sepsis-overall task, the masking model again outperformed all subgroups except for the 35- to 45-year bin. Older groups (older than 55 years) generally saw a larger improvement, with the greatest increase in AUROC seen in the 65- to 75-year group, at 4%. While the surgical and medical ICUs had the same AUROC without IM, the masking model performed better on the surgical ICU.

Brier score trends generally showed similar or improved calibration on including IM features for all outcomes and subgroups. Particularly for P19 sepsis-overall, calibration improved despite external validation.

**Table 2:** Subgroup analysis results for the PhysioNet 2012 Challenge mortality classification task.

			#Samples		Masking (AUROC^a^), mean (SD)		Masking (Brier), mean (SD)		No masking (AUROC), mean (SD)		No masking (Brier), mean (SD)
**Age strata (years)**											
	≤35	268		0.847 (0.74-0.93)		0.057 (0.037-0.079)		0.852 (0.75-0.94)		0.059 (0.040-0.079)	
	35-45	309		0.906 (0.84-0.96)		0.048 (0.031-0.066)		0.880 (0.80-0.95)		0.054 (0.037-0.072)	
	45-55	569		0.878 (0.82-0.93)		0.064 (0.050-0.078)		0.885 (0.83-0.93)		0.064 (0.052-0.077)	
	55-65	708		0.859 (0.82-0.90)		0.074 (0.060-0.090)		0.848 (0.80-0.89)		0.076 (0.063-0.090)	
	65-75	845		0.830 (0.79-0.87)		0.094 (0.079-0.109)		0.822 (0.78-0.86)		0.094 (0.080-0.108)	
	>75	1294		0.801 (0.77-0.83)		0.135 (0.121-0.149)		0.786 (0.75-0.82)		0.135 (0.123-0.149)	
**ICU^b^ types**											
	Coronary care unit	587		0.806 (0.75-0.86)		0.087 (0.069-0.106)		0.807 (0.74-0.86)		0.086 (0.070-0.104)	
	Cardiac surgery unit	780		0.862 (0.79-0.92)		0.035 (0.025-0.046)		0.845 (0.76-0.92)		0.037 (0.028-0.048)	
	Surgical ICU	1192		0.852 (0.82-0.88)		0.094 (0.082-0.107)		0.843 (0.81-0.87)		0.095 (0.083-0.106)	
	Medical ICU	1434		0.801 (0.77-0.83)		0.128 (0.115-0.140)		0.787 (0.76-0.82)		0.129 (0.117-0.141)	

^a^AUROC: area under the curve of the receiver operating characteristic.

^b^ICU: intensive care unit.

**Table 3:** Subgroup analysis results for the PhysioNet 2012 Challenge length of stay classification task.

			#Samples		Masking (AUROC^a^), mean (SD)	Masking (Brier), mean (SD)		No masking (AUROC), mean (SD)		No masking (Brier), mean (SD)
**Age strata (years)**										
	≤35	268		0.862 (0.80-0.92)		0.081 (0.055-0.109)	0.707 (0.61-0.80)		0.108 (0.079-0.138)	
	35-45	309		0.820 (0.71-0.91)		0.060 (0.040-0.081)	0.721 (0.62-0.82)		0.079 (0.057-0.104)	
	45-55	569		0.800 (0.72-0.88)		0.057 (0.042-0.073)	0.712 (0.63-0.79)		0.064 (0.048-0.081)	
	55-65	708		0.797 (0.71-0.87)		0.045 (0.033-0.059)	0.790 (0.72-0.86)		0.054 (0.042-0.068)	
	65-75	845		0.803 (0.72-0.87)		0.047 (0.035-0.060)	0.712 (0.64-0.78)		0.053 (0.042-0.065)	
	>75	1294		0.814 (0.77-0.86)		0.056 (0.046-0.067)	0.747 (0.69-0.80)		0.062 (0.052-0.073)	
**ICU^b^ types**										
	Coronary care unit	587		0.791 (0.73-0.85)		0.086 (0.068-0.105)	0.763 (0.71-0.82)		0.095 (0.078-0.112)	
	Cardiac surgery unit	780		0.890 (0.77-0.98)		0.013 (0.006-0.020)	0.759 (0.60-0.90)		0.018 (0.011-0.025)	
	Surgical ICU	1192		0.812 (0.75-0.87)		0.046 (0.036-0.056)	0.710 (0.64-0.77)		0.056 (0.036-0.056)	
	Medical ICU	1434		0.776 (0.73-0.82)		0.071 (0.060-0.083)	0.682 (0.63-0.73)		0.082 (0.071-0.094)	

^a^AUROC: area under the curve of the receiver operating characteristic.

^b^ICU: intensive care unit.

**Table 4:** Subgroup analysis results for the PhysioNet 2019 Challenge sepsis-overall classification task. A total of 6095 patients did not have intensive care unit type specified, and thus, they were not considered for the corresponding analysis.

			#Samples		Masking (AUROC^a^), mean (SD)		Masking (Brier), mean (SD)		No masking (AUROC), mean (SD)		No masking (Brier), mean (SD)
**Age strata (years)**											
	≤35	1742		0.904 (0.86-0.94)		0.037 (0.029-0.045)		0.893 (0.85-0.93)		0.044 (0.035-0.052)	
	35-45	1949		0.911 (0.88-0.94)		0.041 (0.033-0.049)		0.910 (0.88-0.94)		0.046 (0.038-0.055)	
	45-55	3334		0.920 (0.90-0.94)		0.032 (0.026-0.037)		0.900 (0.87-0.93)		0.037 (0.032-0.043)	
	55-65	4581		0.897 (0.87-0.92)		0.042 (0.037-0.048)		0.886 (0.86-0.91)		0.048 (0.042-0.053)	
	65-75	4768		0.917 (0.90-0.94)		0.039 (0.034-0.043)		0.877 (0.85-0.90)		0.049 (0.043-0.054)	
	>75	3626		0.896 (0.87-0.92)		0.040 (0.034-0.046)		0.888 (0.86-0.91)		0.045 (0.039-0.051)	
**ICU^b^ types**											
	Medical ICU	6923		0.895 (0.88-0.91)		0.044 (0.040-0.048)		0.882 (0.86-0.90)		0.049 (0.045-0.053)	
	Surgical ICU	6982		0.903 (0.89-0.92)		0.041 (0.037-0.045)		0.882 (0.86-0.90)		0.050 (0.046-0.055)	

^a^AUROC: area under the curve of the receiver operating characteristic.

^b^ICU: intensive care unit.

### Simulated Prospective Classification

The last row of Table 1 summarizes the nontemporal evaluation for this task setting. Unlike overall classification, the no masking model outperforms the masking model while keeping almost the same calibration.

Before discussing temporal performances, it is necessary to understand the LOS distribution for each patient category. LOS averaged over the entire cohort was very similar for both P19 hospitals, at 39.77 (SD 22.55) hours and 38.23 (SD 23.27) hours for A and B, respectively. Separating the cohort into patients who eventually develop sepsis and those who don’t shows that patients who develop sepsis spend a longer time in the ICU. For hospital A, septic patients spent 59.54 (SD 57.81) hours on average while nonseptic patients spent 37.87 (SD 13.92) hours. Similarly, for hospital B this was 59.22 (SD 61.90) hours for septic patients and 36.96 (SD 17.72) hours for nonseptic patients. The cohort for both hospitals consisted almost entirely of patients with sepsis after 3 days.

**Figure 4:**
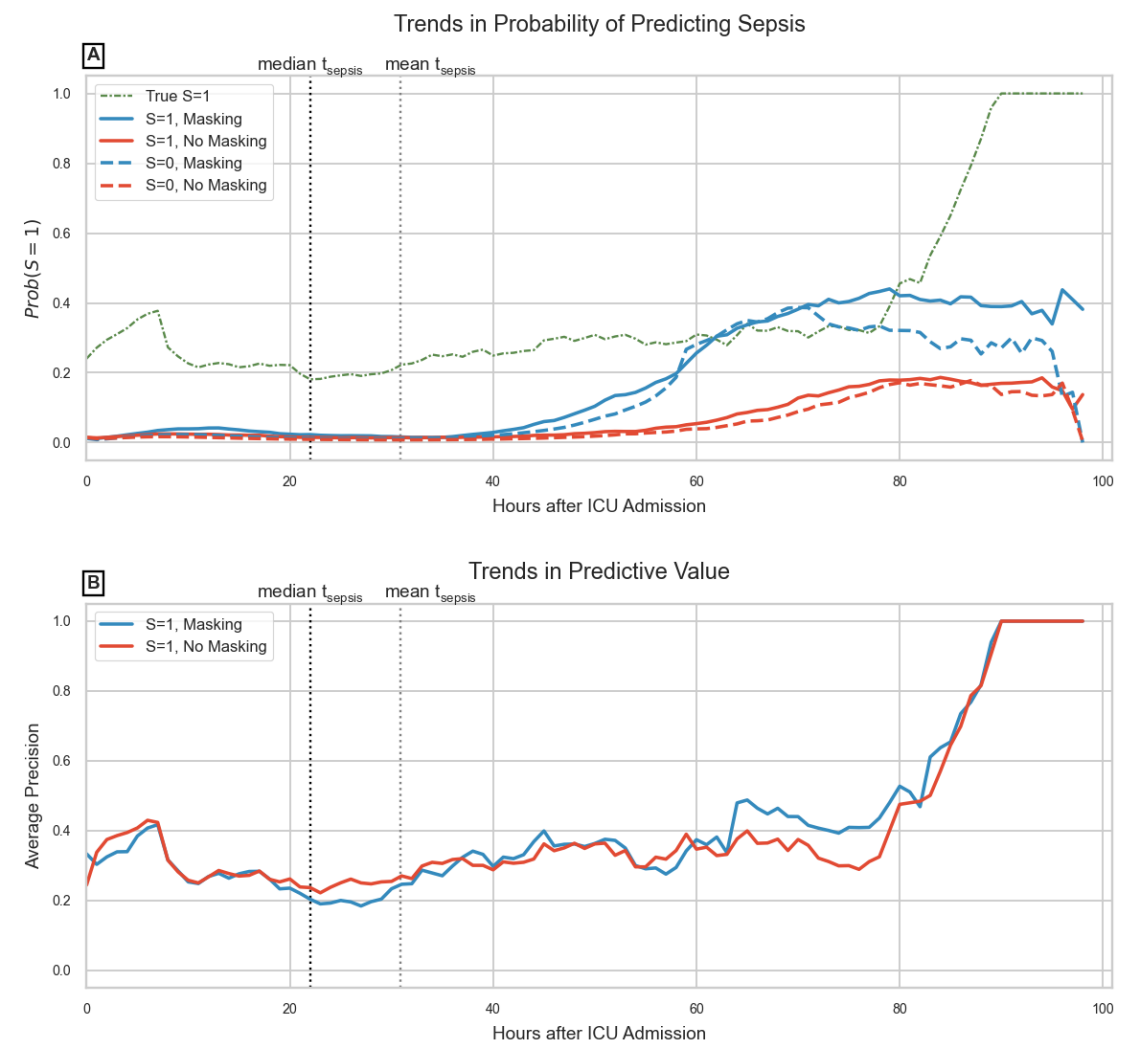
Temporal evaluation for the PhysioNet 2019 Challenge sepsis-frequent task; records corresponding to sepsis are labeled as S=1 while the remainder are S=0: (A) drop in probability of false-positive prediction (S=0) is because after 90 hours, only patients with sepsis remain in the data; (B) this cohort characteristic is learned by the model resulting in perfect predictive value after 90 hours. ICU: intensive care unit.

Temporal evaluation shown in Figure 4B displays almost equal predictive value at each hour over the first 100 hours of ICU admission, with peak predictive value achieved a little over 90 hours. This is likely due to the LOS characteristics of the datasets. Figure 4A shows how model predictions change over time for patients who eventually develop sepsis and those who don’t. We observe a considerable divergence between the curves of masking and no masking models (regardless of sepsis category) a little after 2 days of ICU admission. The same plot also shows trends in the proportion of septic patients at each hour, giving an insight into the expected amount of false alarms or missed diagnoses by each model.

## Discussion

### Principal Findings

Results from the retrospective-overall classification shown in Table 1 were consistent with previous studies [11,13,14], confirming that including even simple representations of health care processes like binary IM features improves performance. This was further reinforced by evaluating the models on a variety of metrics summarizing predictive value and calibration. Model discrimination and predictive value were improved in all cases while keeping the same or better calibration. Results of the P19 sepsis-overall task also confirmed that model generalization in such retrospective tasks is not affected by including IM features, despite interhospital variations. Calibration plots in panel C of Figures 1-3 showed that model reliability was improved for nearly all levels of model certainty, especially for higher predicted probabilities, making the masking model more trustworthy.

Subgroup analysis helped us verify the IM inclusion effect on population subgroups and whether health care process variables encoded information about pathophysiology despite intra- and interhospital variations, justifying their use as proxy biomarkers of patient health. In the P12 mortality subgroup task (Table 2), while the masking model performed better on average in the entire test set, it failed to improve upon the no masking model for certain age groups suggesting that for younger patients, trends in physiological features alone are better predictors of in-hospital death. The masking model was also slightly outperformed by the no masking model in the CCU subgroup, which may be because CCU patients have a very specific set of complications, rendering several laboratory tests unnecessary [48]. For subgroups in P12 LOS (Table 3), however, considerable improvements in AUROC for younger age groups were observed, suggesting laboratory tests conducted were important indicators to estimate whether a patient will spend more or less than 3 days in the ICU. The CCU again saw only a slight improvement, probably due to a generally earlier diagnosis relative to other ICUs. Overall, for both P12 outcomes, younger age groups and the cardiac surgery recovery unit had the highest AUROCs achieved by masking models.

For subgroups in the P19 sepsis-overall task (Table 4), older age groups generally saw greater benefit on IM inclusion. Sepsis is known to be associated with age, which may in turn prompt physicians to order relevant tests earlier in the patient’s ICU stay [49]. The surgical ICU again saw a greater improvement in AUROC over the medical ICU, while the model had almost equal performance for both ICUs using only physiological features. This task also evaluated model performance and effect of IM features on model generalization, since the subgroups were made using data from a distinct hospital. These results suggest that, at least in retrospective task settings, health care process variables do not hinder model generalization and models trained using these variables can adequately learn the relation of IM features to the underlying condition without being affected by interhospital variations.

Calibration indicated by the Brier score showed that the model actually learns to output better probabilities on including health care process variables.

### Relationship With Prior Work

Perhaps the study most similar to this work was by Sharafoddini et al [50], which examined whether missing indicator features are informative. The study performed extensive data analysis and evaluated logistic regression and tree-based models trained with and without missing indicators to assess any difference in discriminative ability. Their results demonstrated improved model performance upon IM inclusion, and feature selection methods reinforced the importance of IM variables. While this work is similarly motivated in its goal to objectively assess IM features, there are some essential differences. We focused on several outcomes of interest as opposed to mortality only, as discussed earlier. We also provided comprehensive evaluation through multiple metrics, assessing not only overall discrimination but also hourly discrimination and model calibration. Subgroup analysis and evaluation of model generalization on a distinct patient population further contribute to the novelty of this work. Previous studies did not evaluate their model’s performance on ICU population subgroups, instead assuming similar performances across patients [9,13,14]. We showed that discrimination varies between strata as does the extent of improvement brought by including IM features. Finally, we used a sequential deep learning model (GRU) as opposed to the models used in Sharafoddini et al [50], since RNN variants have been popular choices to model EHR data and often use IM features to improve performances [13,14].

Temporal trends in probability of predicting sepsis shown in Figure 4A confirm previous findings by Sharafoddini et al [50] that indicators become increasingly important from the second day onward in the ICU. But this is arguably too late, since patients who eventually developed sepsis had a higher variance in LOS, many becoming septic early on in their ICU stay. While including IM features results in better model performance overall, it also falsely identifies nonsepsis patients as susceptible (false positives) in the near future, leading to several false alarms. In the PhysioNet 2019 Challenge, the utility score metric applied a minimal penalty for false positive predictions, while also leading to earlier and greater true positives, perhaps explaining the extensive use of IM features in proposed models. But alarm fatigue is a known issue in ICU early warning scores, and false positives cannot be ignored [51]. When performance on predicting the absence of sepsis (true negatives) is not considered, the net predictive value gets balanced out, as shown in Figure 4B. Also, unlike previous studies, which relied on end-of-day outcome prediction or thresholded decision outputs for evaluation, we relied exclusively on hourly probabilities and visualized its trends with time, which may be used to understand a model’s clinical utility more comprehensively [27,42].

It is important to understand that IM feature effectiveness varies based on the outcome of interest, whether they are applied for retrospective or prospective tasks and even on population subgroups. With IM features now being used for a variety of tasks including classification, prediction, and even imputation, models relying on these may further propagate preexisting biases in health care processes.

### Limitations

A limitation of this study was using data from the same country, in this case the United States. Practices and case-mix vary by country. Physician attitudes to uncertainty (which may influence test ordering and drug prescription) may also be affected by resource limitations and even by cultural factors [24]. This requires verifying masking model generalizability on data from different parts of the world. Efforts have been made to standardize test ordering behavior but guidelines are followed to varying extents depending on patient histories, comorbidities, and the physician in charge [26,52].

The datasets we used were observational, with no information regarding the context in which laboratory tests were ordered or which patients were transfers from other ICUs. The latter leads to the problem of lead-time bias, which may be reflected in the data as unexpected adverse outcomes for certain patients [53]. We also evaluated IM feature effectiveness on only one model type, GRU (an RNN variant). While we selected this because of its common use in prior work, different models may learn IM representations differently [38].

Critical care EHRs are also a specific subtype of general EHRs, since they consists only of inpatients with serious conditions. A more general EHR dataset that includes outpatients may result in different health care process observation patterns and reveal interesting effects on predictive models [23]. Finally, clinical best practices change over time, in turn affecting which tests are performed and how often. This is part of the larger problem of dataset shift in machine learning, and it remains to be seen how this would affect clinical models relying on health care process features.

### Conclusion and Future Work

With increasing use of observational EHR data for machine learning model development, there has been an increase in the number of studies claiming clinical utility of proposed models, many relying on variables representative of health care processes. In this study, we addressed questions regarding the effect of using health care process features on machine learning model performance and generalizability. By separating commonly used task settings into 2 subtypes, retrospective and (simulated) prospective, we made an important distinction concerning possible clinical utility of models. We framed all our results using multiple evaluation metrics while also analyzing external validation performances for all tasks by using data from a geographically distinct hospital.

This study demonstrated the usefulness of IM features in retrospective task settings on various outcome labels. Notably, we found that machine learning model generalization and calibration are not adversely affected on using health care process variables even when externally evaluated. However, the extent of improvement may depend on different patient and in-hospital factors such as age or ICU type. Our research indicated that these features provide better information for certain subgroups than others, and IM variables are better predictors of administrative outcomes like length of stay than mortality or sepsis. Results also showed that, at least for a sequential deep learning model, using simple binary missingness indicators for simulated prospective sepsis classification did not add any benefit over a model relying on patient pathological features only.

Our findings suggest that the suitability of using IM features in machine learning models may vary based on the outcome of interest, subgroup of application, task setting (retrospective or prospective), and differences in clinical practice between training data and test data. Class imbalances and nature of outcome have an intense impact on expected performance improvements on IM feature inclusion. In application, the subgroup of a patient and deviation in model performance from its expectation also need to be considered while estimating the uncertainty of a prediction. Also, while ultimately machine learning models aim to lend themselves to use as continuous monitoring bedside tools, using IM features does not seem to add any prominent improvement over not using them in that setting. Finally, using IM means using clinical practice variables in a model, so different missingness rates and missingness patterns need to be properly contextualized to understand model performance differences between train and test environments. Biased observations in one dataset (due to practice or even hospital resource variations) may have a substantial effect on model discriminations and calibration in another dataset.

There are several ways to extend this study. Future work may (1) focus on verifying model performance and generalization changes by using data from multiple countries, (2) focus on using different types of models and analyze how differently learned representations of missingness affect performance, or (3) study how health care process features may be used for multilabel classification.

## Appendix

Multimedia Appendix 1 Description of the sepsis label in PhysioNet 2019 Challenge.

Multimedia Appendix 2 Further data and result analysis.

## Abbreviations

AUROC: area under the curve of the receiver operating characteristic
CCU: coronary care unit
EHR: electronic health record
GRU: gated recurrent unit
ICU: intensive care unit
IM: informative missingness
LOS: length of stay
MIMIC II: Multiparameter Intelligent Monitoring in Intensive Care
P12: PhysioNet 2012 Challenge dataset
P19: PhysioNet 2019 Challenge dataset
RNN: recurrent neural network
Sepsis-3: Third International Consensus Definitions for Sepsis and Septic Shock
