# Supplementary material for: On Missingness Features in Machine Learning Models for Critical Care: Observational Study
Source: JMIR Med Inform. 2021 Dec 8;9(12):e25022. doi: 10.2196/25022 (PMC8701717; doi:10.2196/25022)
Supplement: Multimedia Appendix 1 [file medinform_v9i12e25022_app1.pdf]

## Multimedia Appendix 1: Description of the sepsis label in P19

Here, we provide a description of the sepsis label as defined by the organizers of the Physionet 2019 challenge ([29] in the main manuscript).

A binary sepsis label (*S*) was defined by first identifying the time of sepsis onset. This was denoted by $\boldsymbol{t}_{\mathbf{sepsis}}$.

To define $t_{sepsis}$ the authors of [29] define two additional supporting time points: $\boldsymbol{t}_{\boldsymbol{suspicion}}$ and $\boldsymbol{t}_{\boldsymbol{SOFA}}$.

### $t_{suspicion}$

$t_{suspicion} = \min(t_{IV}, t_{BC})$ *iff* condition 3 and one of conditions 1 and 2 are satisfied;

| | | |
|---|---|---|
| **Cond. 1** | $t_{IV} \leq t_{BC} \leq t_{IV} + 24$ hours | ~ *If IV administered first* |
| **Cond. 2** | $t_{BC} \leq t_{IV} \leq t_{BC} + 72$ hours | ~ *If BC obtained first* |
| **Cond. 3** | IV administration for 72 consecutive hours | |

Where;
$t_{IV}$ is the time of administration of intravenous antibiotics and,
$t_{BC}$ is the time of acquirement of blood cultures.

### $t_{SOFA}$

$t_{SOFA}$ is the time of occurrence of organ failure as defined by a 2-point rise in SOFA score within a 24-hour time period.

### $t_{sepsis}$

Finally, from the above two definitions,
$t_{sepsis} = \min(t_{suspicion}, t_{SOFA})$ if the following condition is satisfied;

**Cond.** $t_{suspicion} - 24 \text{ hours} \leq t_{SOFA} \leq t_{suspicion} + 12$ hours

## Sepsis Label (S)

Finally, the sepsis label was shifted by 6 hours.
**S = 1** if $t \geq t_{sepsis} - 6$ hours and
**S = 0** if $t < t_{sepsis} - 6$ hours
