# Supplementary material for: On Missingness Features in Machine Learning Models for Critical Care: Observational Study
Source: JMIR Med Inform. 2021 Dec 8;9(12):e25022. doi: 10.2196/25022 (PMC8701717; doi:10.2196/25022)
Supplement: Multimedia Appendix 2 [file medinform_v9i12e25022_app2.pdf]

Multimedia Appendix 2: Further Data and Result Analysis

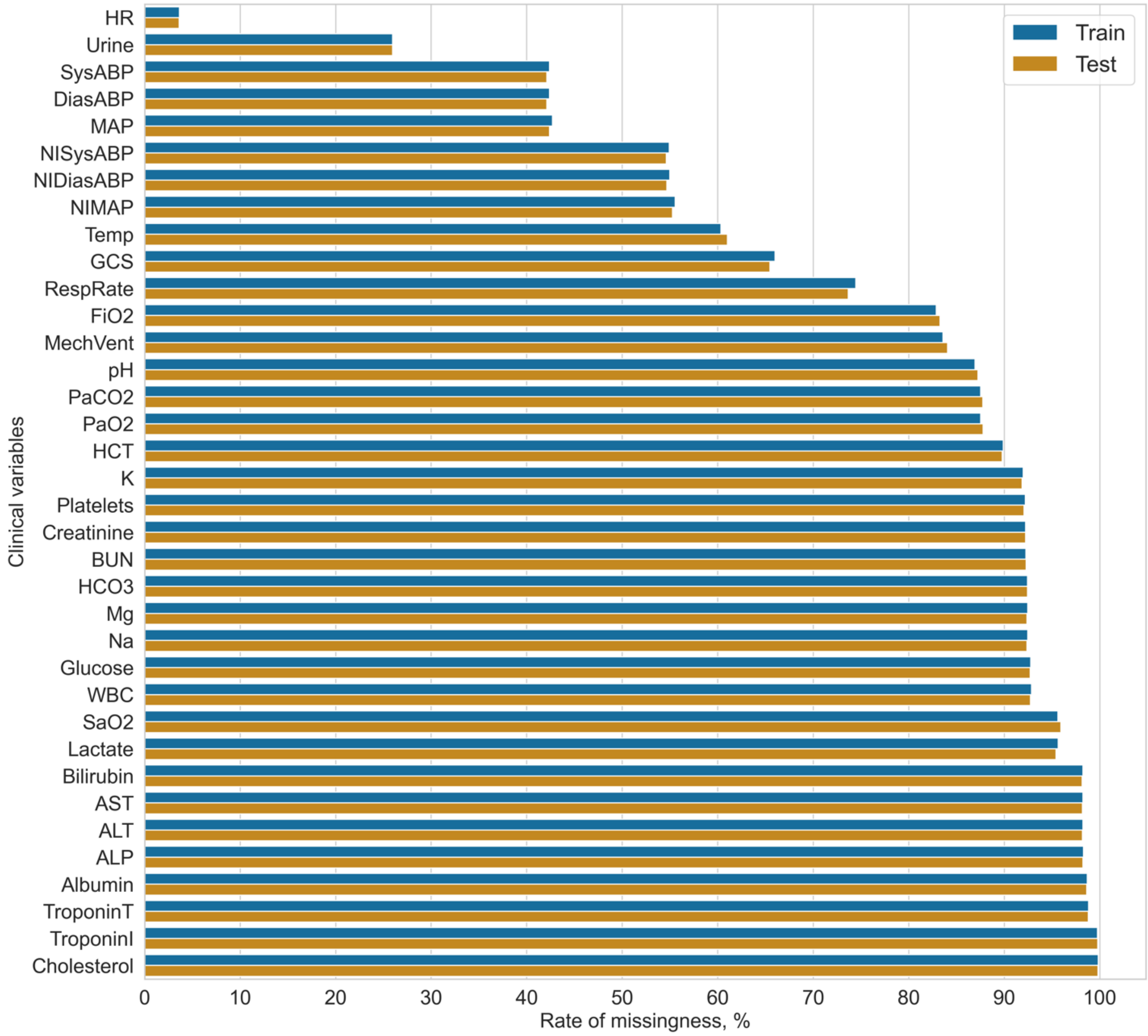

Figure S1. All clinical variables with rates of missingness for the **P12** training and testing sets. Rate of missingness is the average rate at which a variable is missing across all patients. Since all data in P12 was from the same hospital, missing rates are very similar.

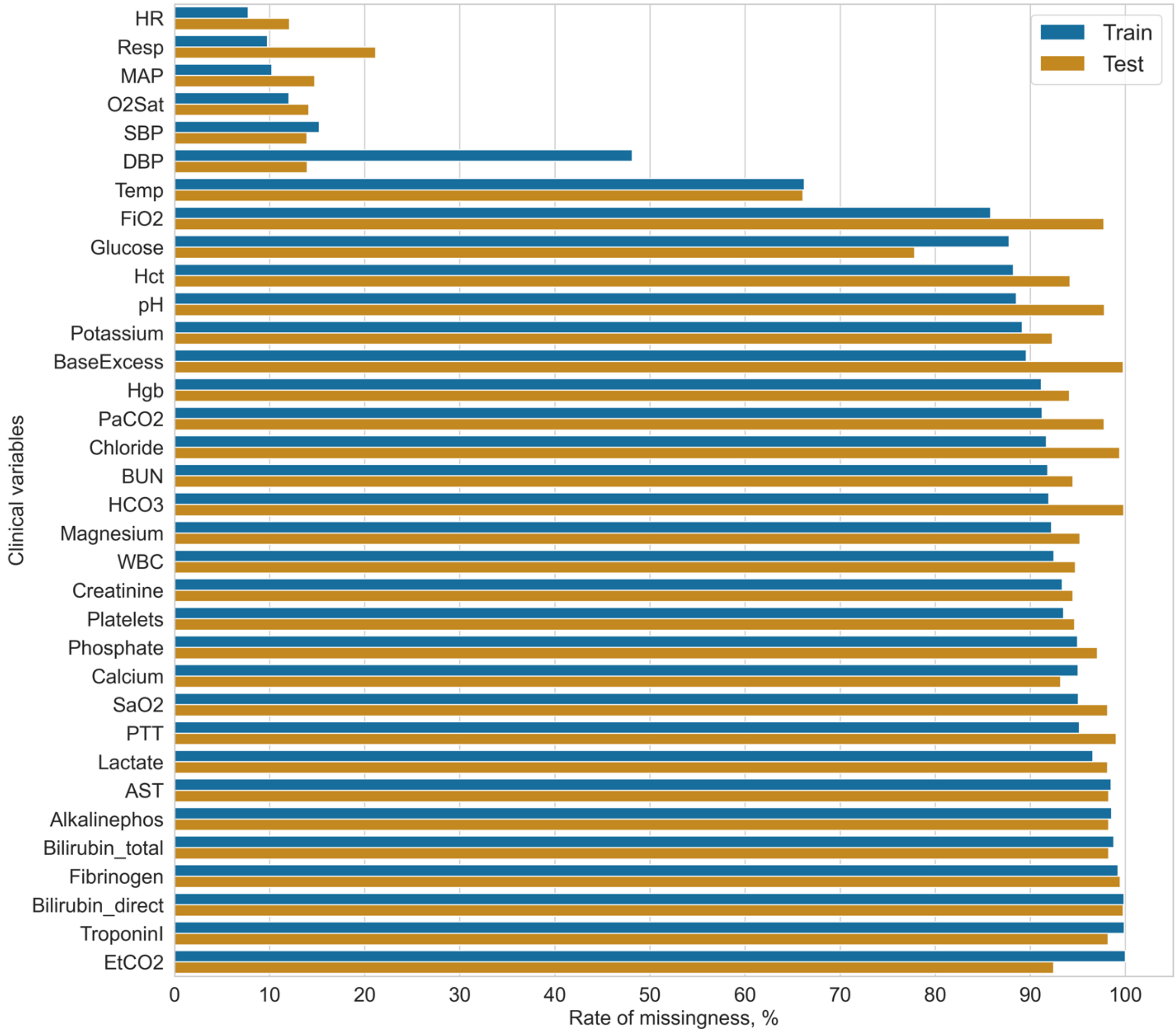

Figure S2. All clinical variables with rates of missingness for the **P19** training and testing sets. Rate of missingness is the average rate at which a variable is missing across all patients. Rates of missingness differ since each set is from distinct hospitals.

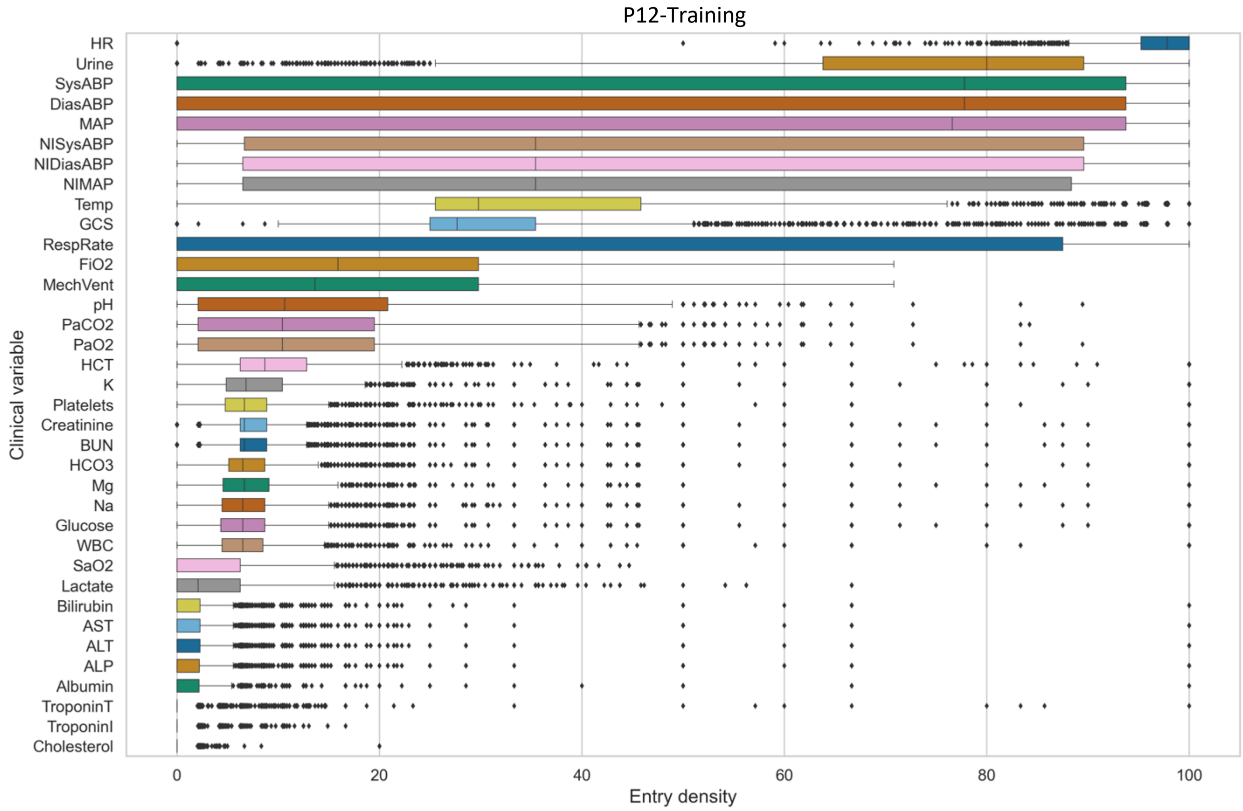

Figure S3. Distribution of entry densities (fraction of observations in a patient's ICU stay) for each variable for the **P12** training set. Entry density is the distribution of the per-patient average observation rate. P12 testing set showed a very similar distribution.

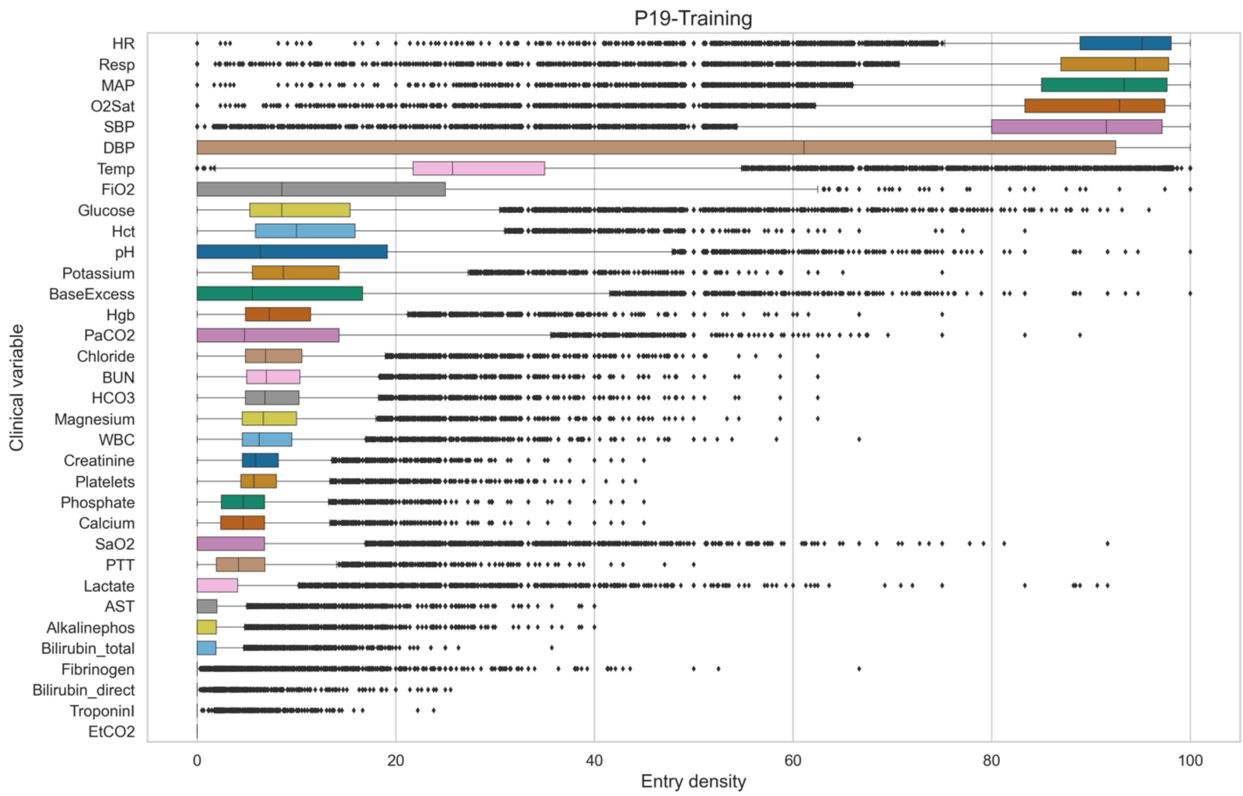

Figure S4. Distribution of entry densities (fraction of observations in a patient's ICU stay) for each variable for the **P19** training set (Hospital A). Entry density is the distribution of the per-patient average observation rate.

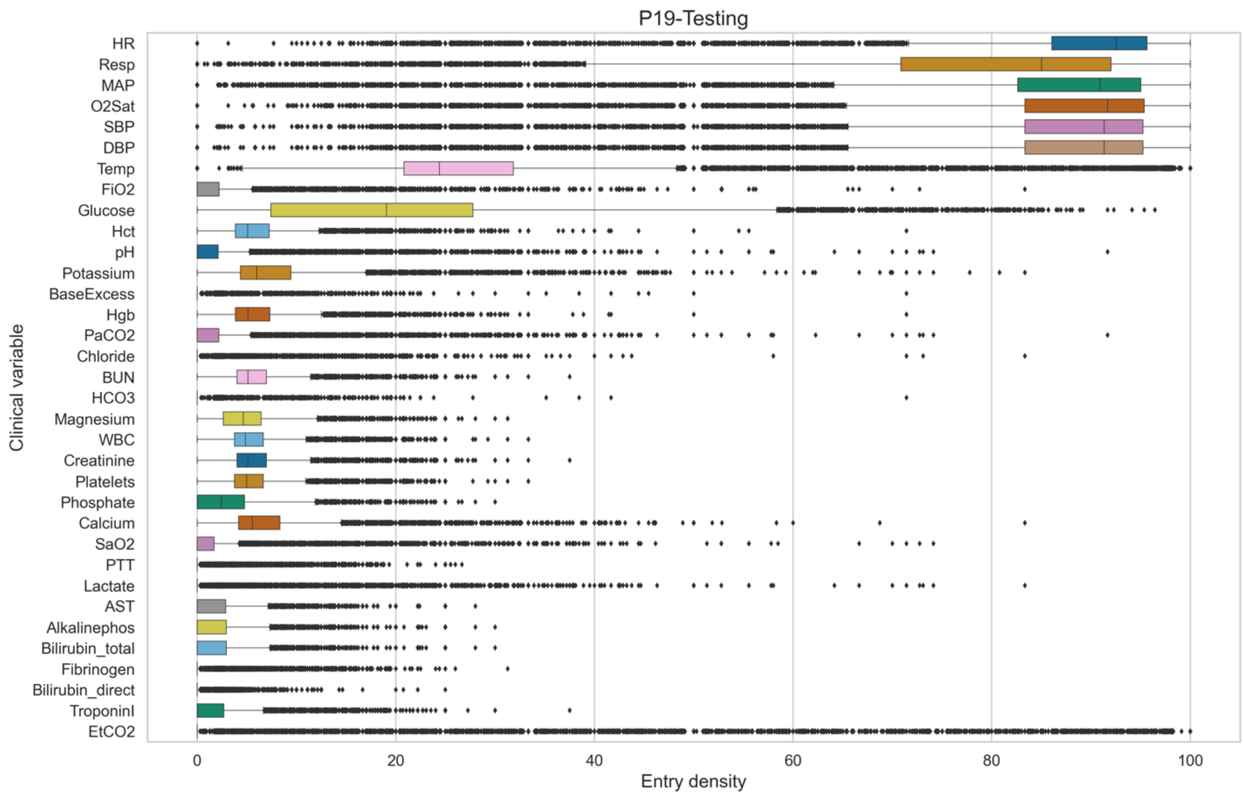

Figure S5. Distribution of entry densities (fraction of observations in a patient's ICU stay) for each variable for the **P19** testing set (Hospital B). Entry density is the distribution of the per-patient average observation rate.

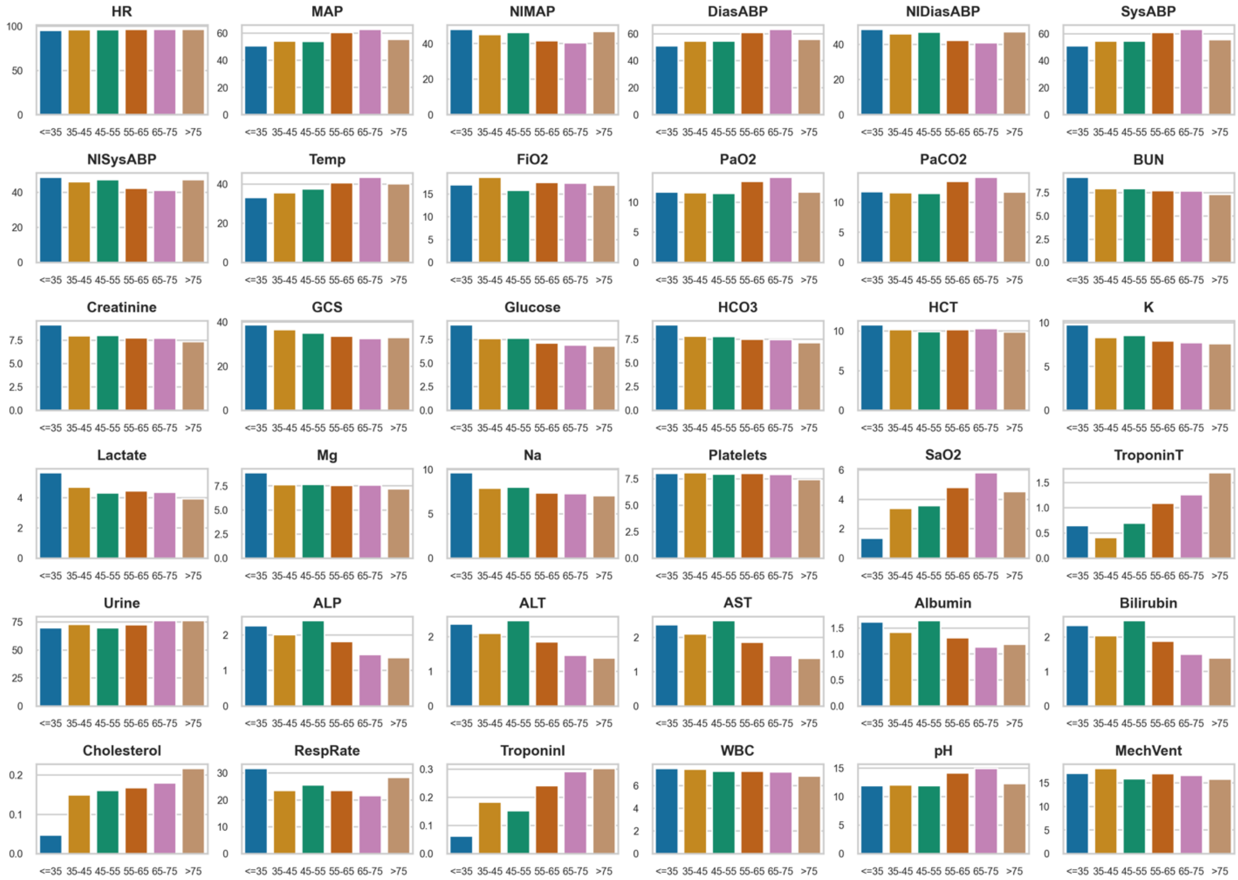

Figure S6. Variable observation rates by age group for the **P12** training set.

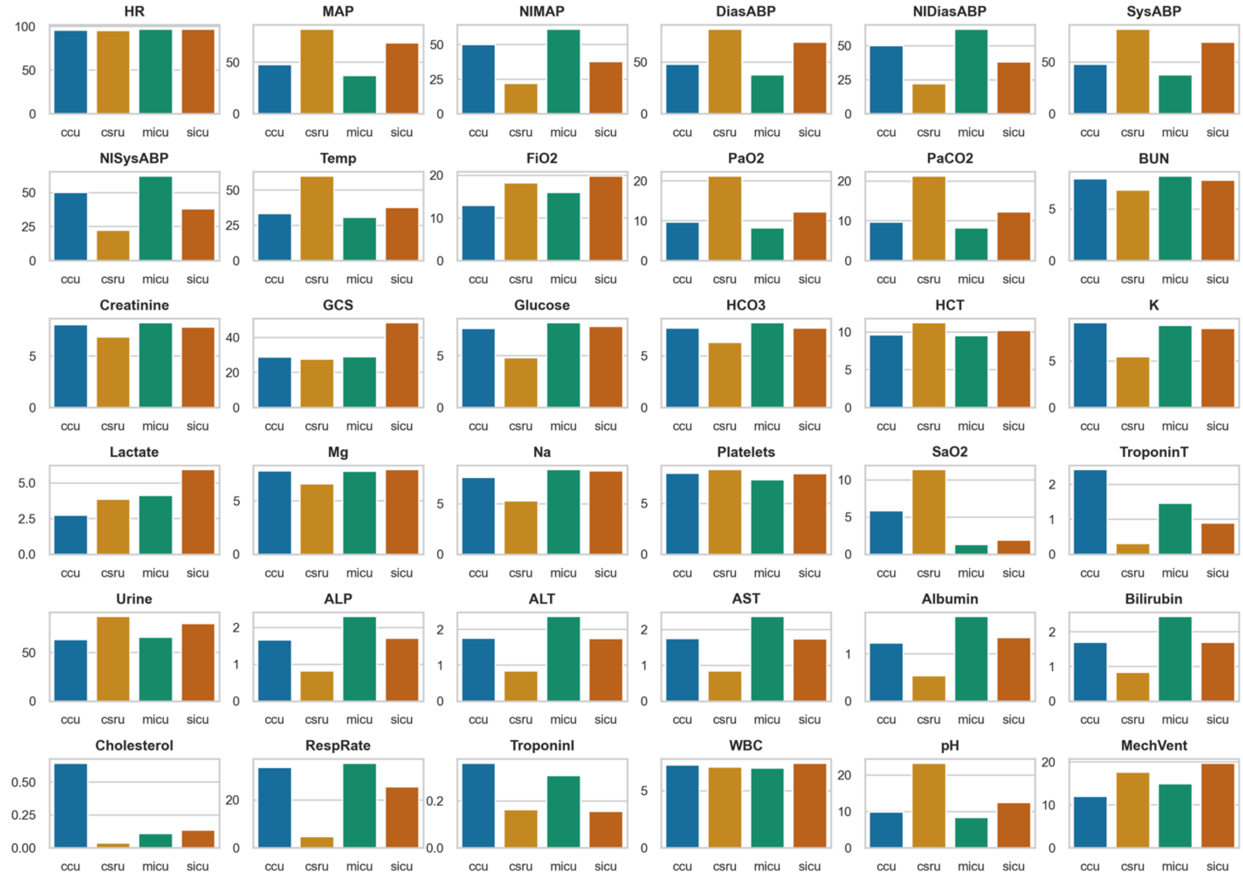

Figure S7. Variable observation rates by ICU types for the **P12** training set.

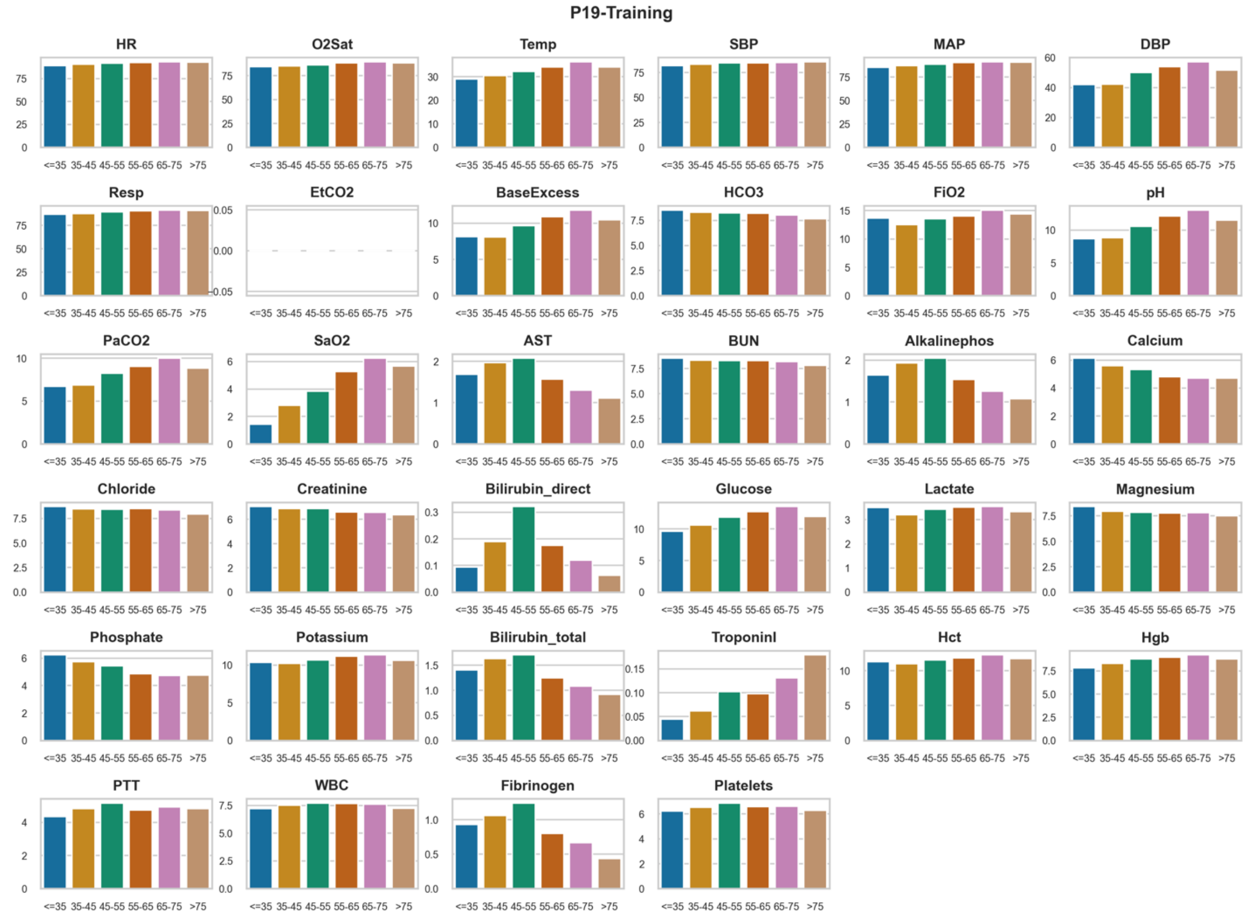

Figure S8. Variable observation rates by age group for the **P19** training set (Hospital A).

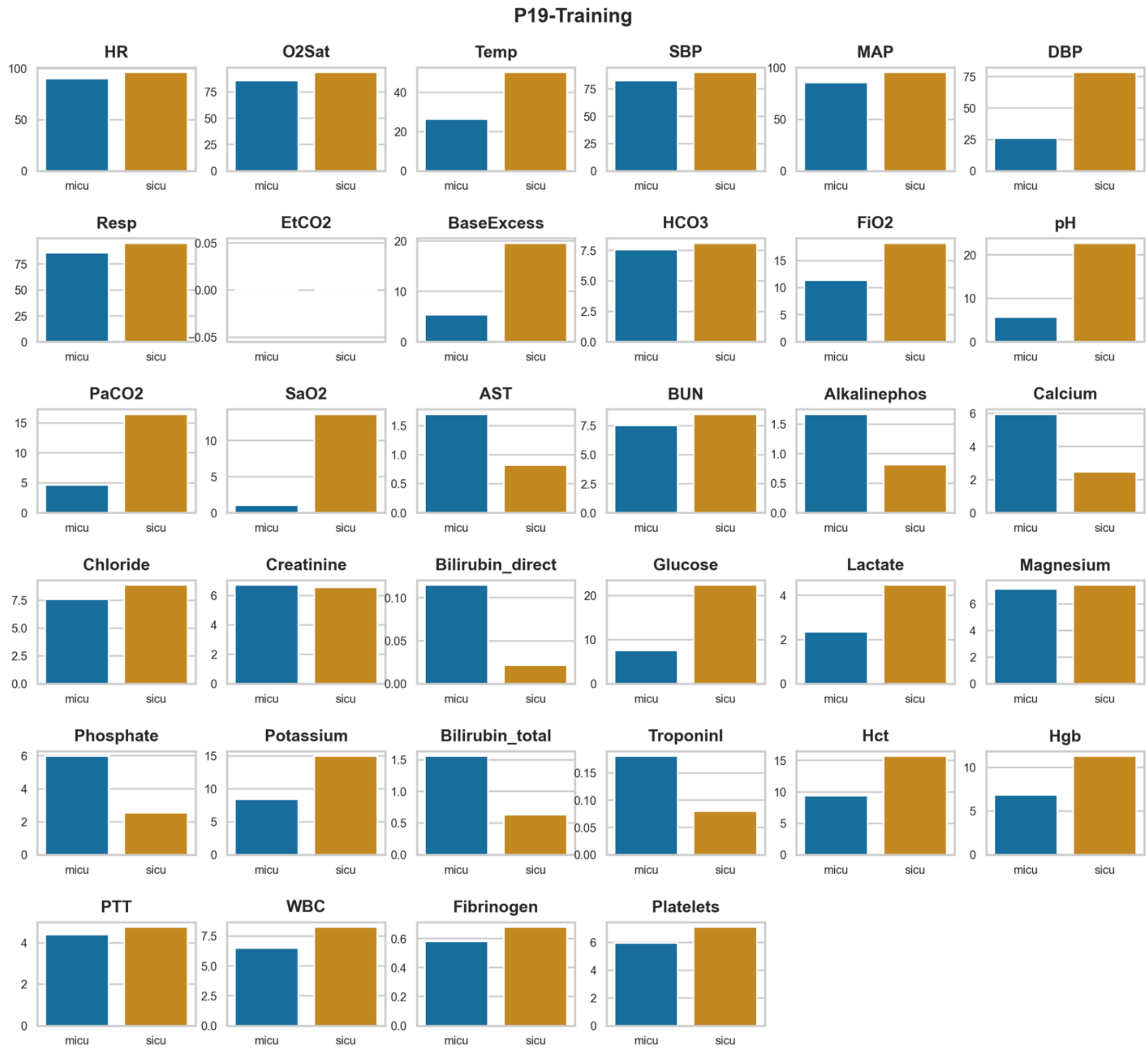

Figure S9. Variable observation rates by ICU types for the **P19** training set (Hospital A).

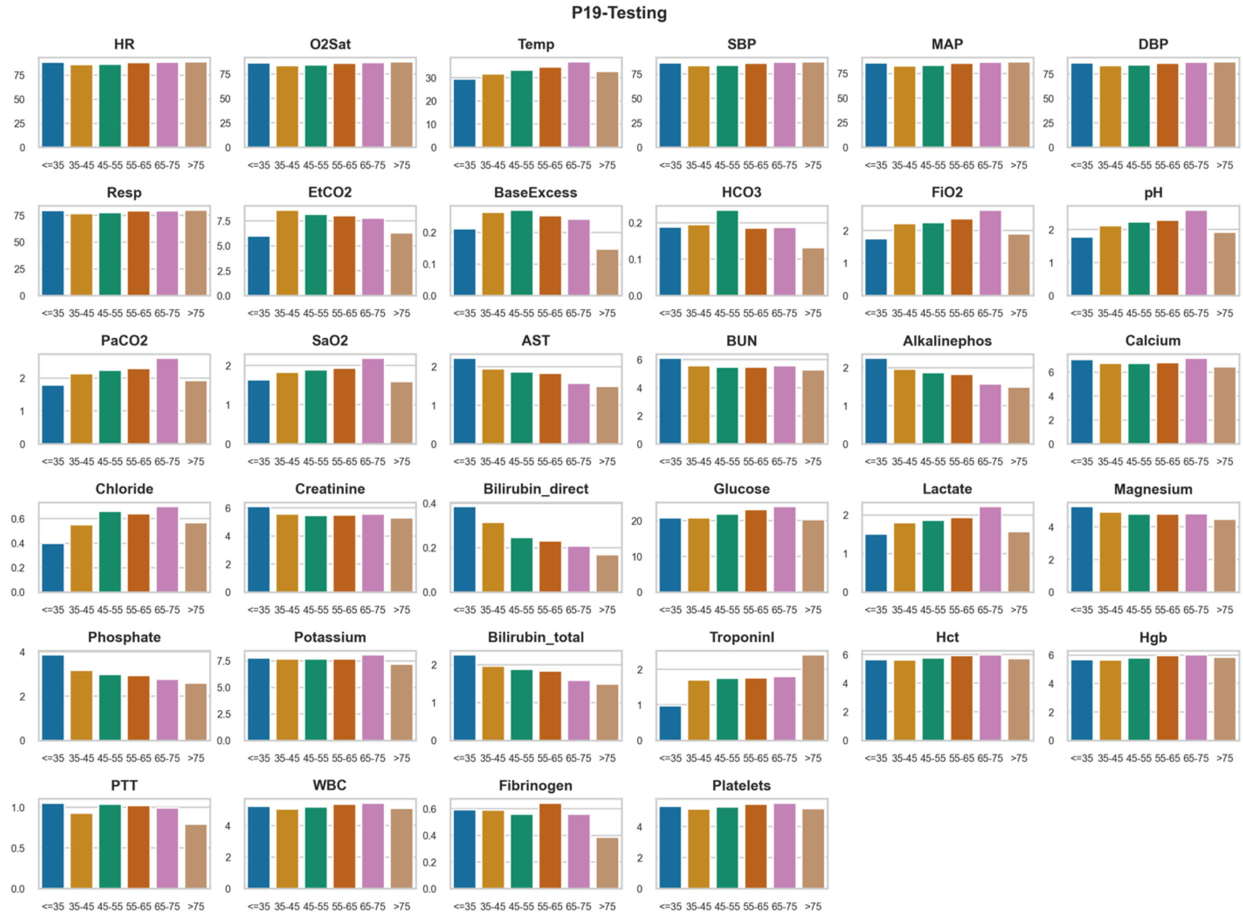

Figure S10. Variable observation rates by age group for the **P19** testing set (Hospital B).

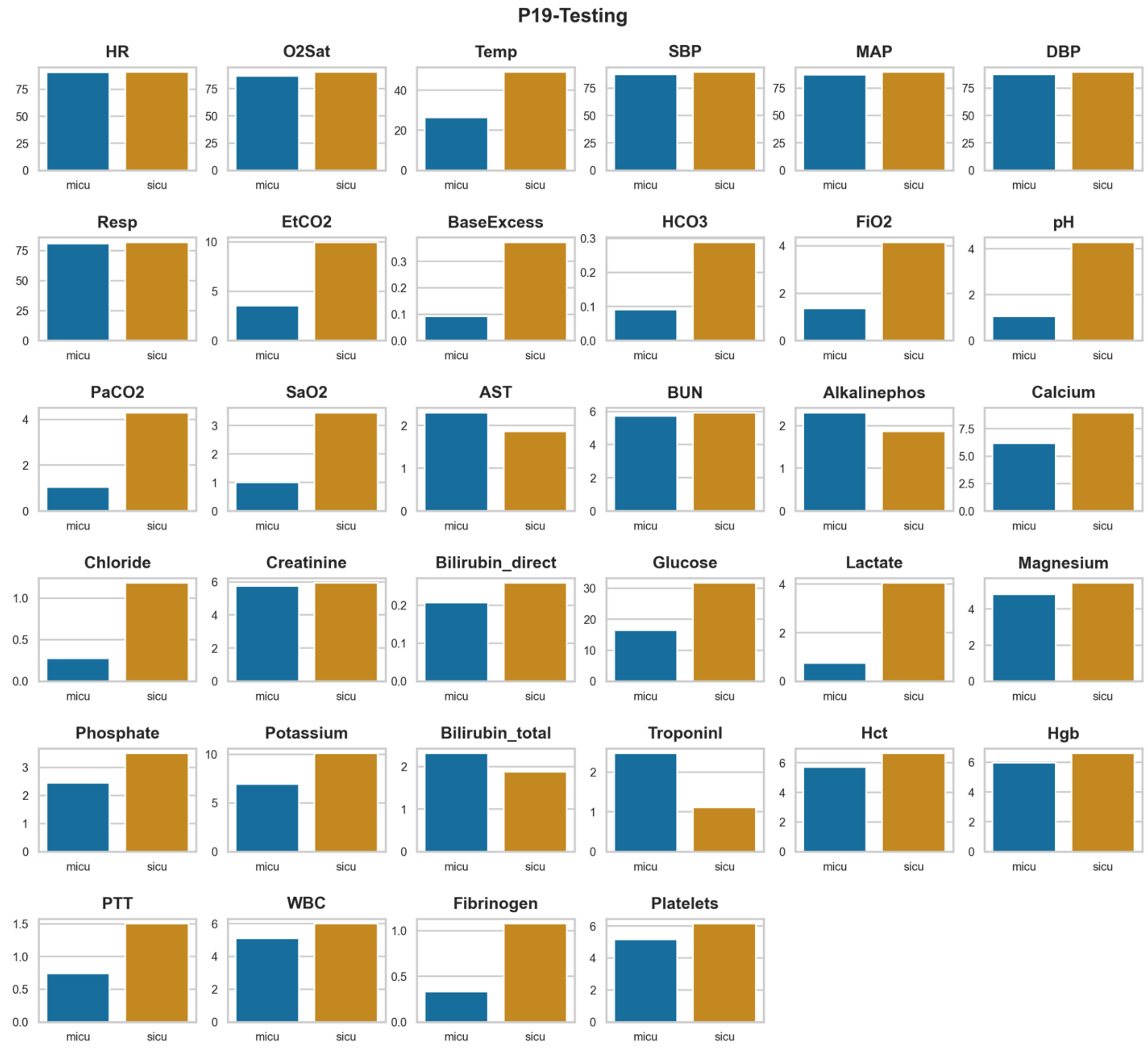

Figure S11. Variable observation rates by ICU types for the **P19** testing set (Hospital B).

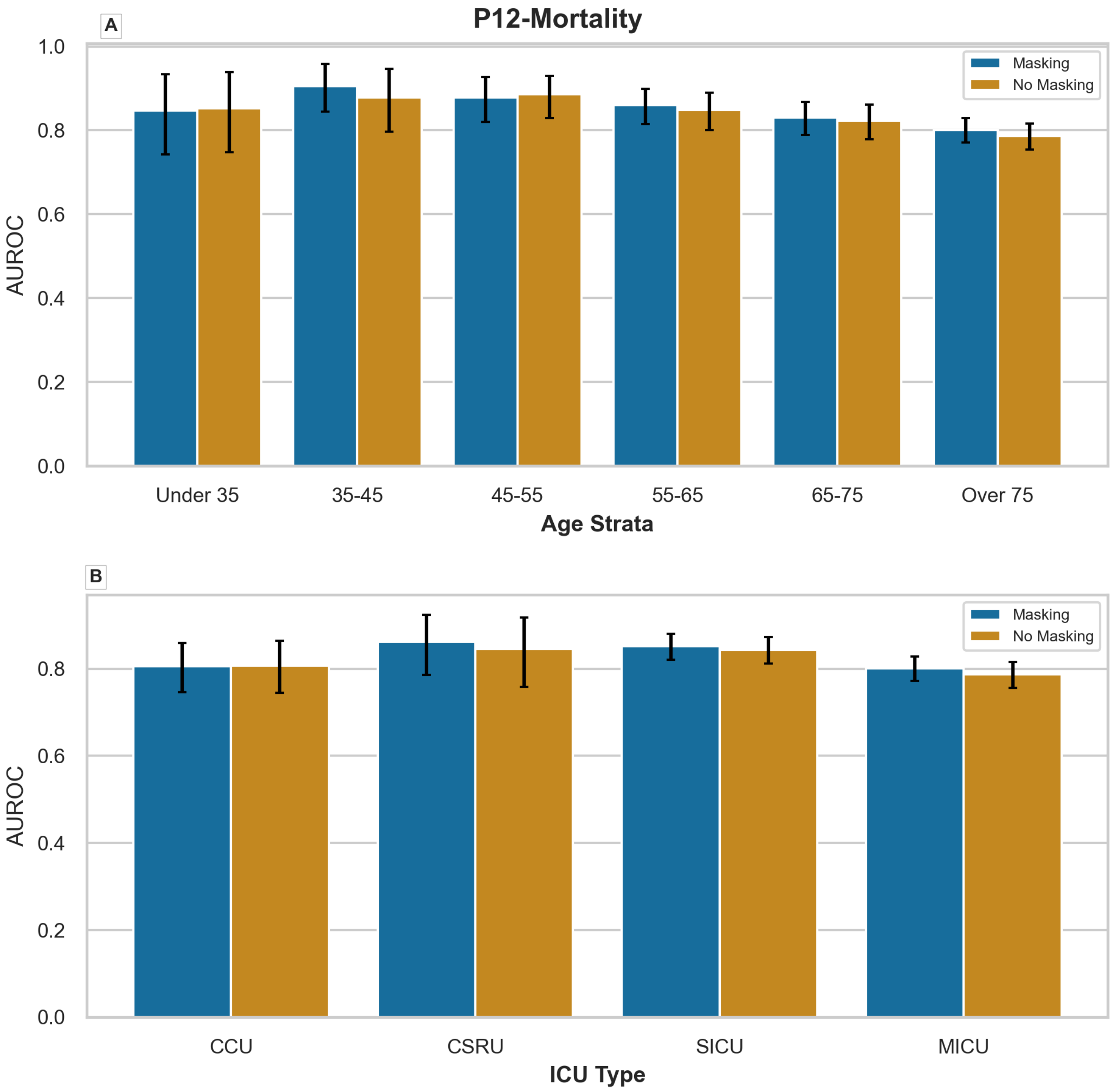

Figure S12. Subgroup Analysis results for the **P12** Mortality Classification task. Error bars represent 95% confidence intervals obtained by bootstrapping. Numerical values are available in Table 2 in the main manuscript.

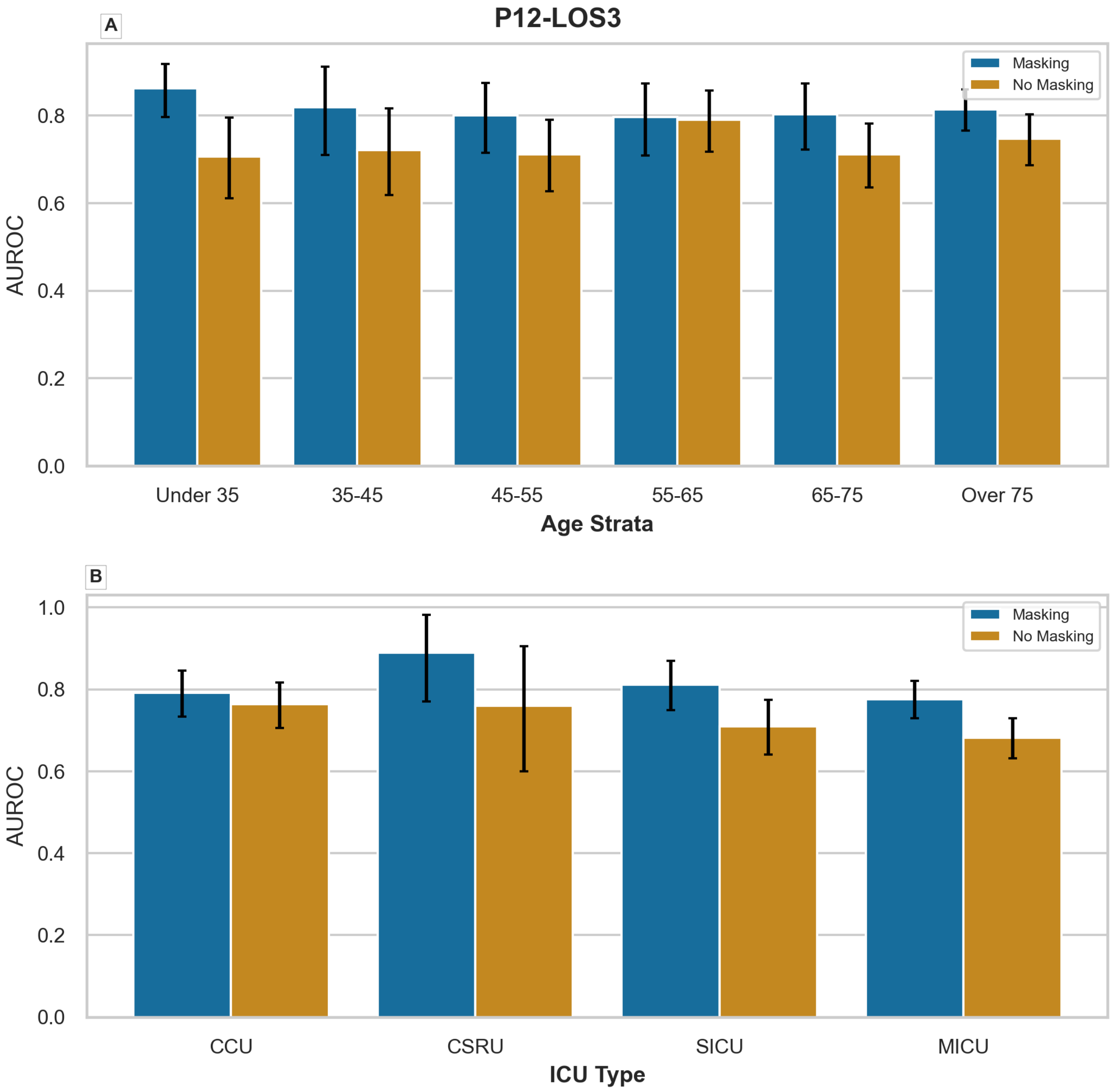

Figure S13. Subgroup Analysis results for the **P12** LOS Classification task. Error bars represent 95% confidence intervals obtained by bootstrapping. Numerical values are available in Table 3 in the main manuscript.

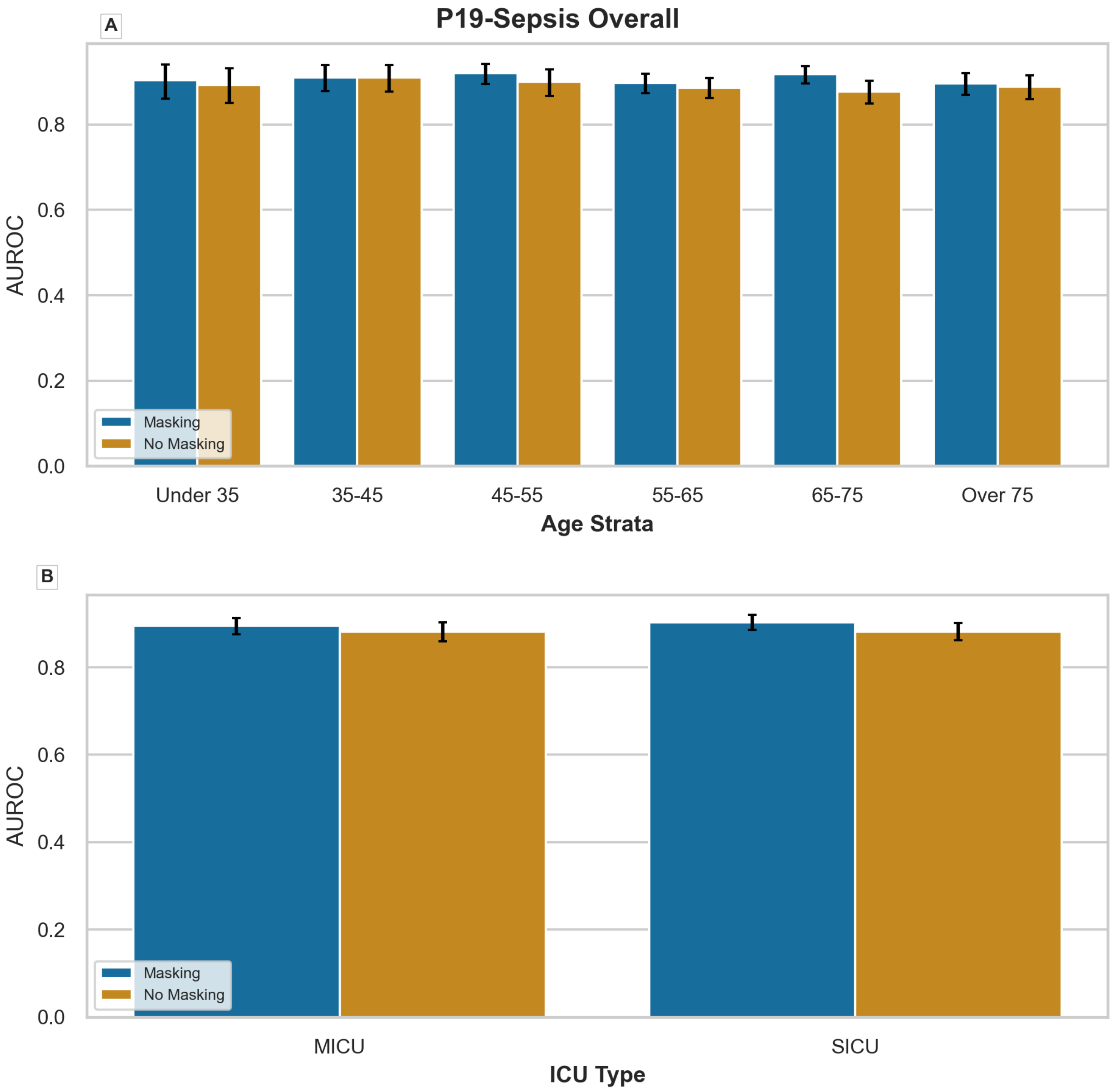

Figure S14. Subgroup Analysis results for the **P19** Sepsis-Overall Classification task. Error bars represent 95% confidence intervals obtained by bootstrapping. Numerical values are available in Table 4 in the main manuscript.
